# Lessons from the slide preparation: A new species of *Kaluginia* (Diptera, Chironomidae) from China

**DOI:** 10.3897/zookeys.1281.193732

**Published:** 2026-06-04

**Authors:** Yiyi Wang, Chao Song, Xin Liu, Xin Qi

**Affiliations:** 1 Zhejiang Key Laboratory for Restoration of Damaged Coastal Ecosystems, Zhejiang International Science and Technology Cooperation Base for Biomass Resources Development and Utilization, School of Life Sciences, Taizhou University, Zhejiang, Taizhou 318000, China Zhejiang Key Laboratory for Restoration of Damaged Coastal Ecosystems, Zhejiang International Science and Technology Cooperation Base for Biomass Resources Development and Utilization, School of Life Sciences, Taizhou University Taizhou China

**Keywords:** Boreoheptagyiini, Chironomidae, *
Kaluginia
*, morphology, non-biting midges, slide preparation, taxonomy

## Abstract

*Kaluginia wangi***sp. nov**., collected from China, is described and illustrated. The species belongs to the tribe Boreoheptagyiini (Diamesinae). The genus *Kaluginia* is newly recorded from China. Phylogenetic analyses based on five molecular markers (18S, 28S, CAD, COI-5p, and COI-3p) confirm the new species belongs to a highly supported clade together with the known species *Kaluginia lebetiformis*. Morphologically, the two species are somewhat similar but can be distinguished by the number of antennal flagellomeres, and the structure of the hypopygium. Through careful slide preparation of the holotype and integrated morphological and molecular cross-validation, this study revealed that variations in slide-mounting techniques can produce morphological artifacts, thereby directly affecting taxonomic conclusions. These findings highlight that for taxa characterized by fine morphological structures, meticulous slide preparation and thorough verification are essential for ensuring robust taxonomic outcomes.

## Introduction

The family Chironomidae, commonly known to comprise non-biting midges, is a globally distributed group of dipteran insects found on all continents, including Antarctica (Ashe and O’Connor 2009). They are renowned for their exceptional adaptability to extreme environments, such as cold (Lencioni et al. 2015; Kozeretska et al. 2021), desiccation (Gusev et al. 2014), and even outer space (Gusev et al. 2010). With over 6300 valid species described worldwide (Lin et al. 2022), chironomids are among the most common and species-rich groups of organisms in freshwater and semi-aquatic habitats.

Within Chironomidae, the subfamily Diamesinae is relatively species-poor, with most species inhabiting cold lotic or oligotrophic lentic environments. They are distributed globally, with the exception of Antarctica. The subfamily is divided newly into four tribes— Diamesini, Boreoheptagyiini, Heptagyini and Lobodiamesini— comprising 27 genera (Brundin 1966; Ashe and O’Connor 2009; Semenchenko et al. 2024). The monophyly of Diamesinae has been confirmed by molecular phylogenetic studies, although the current sampling remains biased toward Southern Hemisphere taxa (Cranston et al. 2010, 2012; Makarchenko et al. 2017, 2018, 2020; Semenchenko et al. 2024).

In this study, we describe *Kaluginia wangi* sp. nov. based on three adult males collected from China. In order to definitively ascertain the phylogenetic placement, we employed an integrative framework. We reconstructed the phylogeny of the subfamily Diamesinae using Bayesian inference (BI) and maximum likelihood (ML) analyses, incorporating our newly generated sequences with typical molecular data from public databases. Beyond the taxonomic description, we also highlight a methodological issue encountered during this study: the holotype, when prepared under different slide-mounting conditions, exhibited distinct morphological appearances of the hypopygium. This observation prompted us to examine the role of slide-preparation techniques in morphological taxonomy, a factor often overlooked yet potentially critical for accurate species delimitation in Chironomidae. Our primary objectives were to formally describe and illustrate the species, determine its phylogenetic position within Diamesinae, and discuss the implications of slide preparation for morphological taxonomy.

## Material and methods

Specimens were prepared on microscope slides following the method described by Sæther (1969). All color descriptions were based on specimens preserved in alcohol prior to permanent mounting in Euparal. Photographs of adult specimens were acquired primarily with an SZ680 zoom stereomicroscope supplemented with a BK6000 biological microscope for detailed imaging of certain organs. The morphological nomenclature is based on Sæther (1980), and the abbreviations for measured parts conform to Sæther (1980). Type specimens were deposited at the College of Life Science, Taizhou University, Taizhou, China (TZU).

To obtain the standard DNA barcode, genomic DNA was first isolated from the adult head and thoracic tissues following the protocol described by Frohlich et al. (1999). The PCR products were verified on a 1.0% agarose gel, purified, and sent for sequencing on an ABI 3730XL capillary sequencer (Beijing Genomics Institute). The resulting sequence chromatograms were assembled and edited using BioEdit 7.2.5 (Hall 1999).

Five molecular markers were used in this study, including two nuclear ribosomal genes (18S and 28S), one nuclear protein-coding gene (CAD), and the mitochondrial COI gene, which included both the 5' and 3' regions (COI-5p and COI-3p). All primer sequences, orientations, and references used for amplification are listed in Suppl. material 1. Published sequences of additional Diamesinae genera were retrieved from public databases and incorporated into the dataset along with newly generated sequences (Suppl. material 2). The protein-coding genes CAD and COI were aligned using ClustalW in Mega X (Kumar et al. 2018), whereas the ribosomal genes 18S and 28S were aligned and trimmed using TrimAl (Capella-Gutiérrez et al. 2009).

Phylogenetic relationships were reconstructed using both maximum likelihood (ML) and Bayesian inference (BI) methods. For the ML analysis, IQ-TREE 3.0.1 (Wong et al. 2025) was employed under a partition scheme separating 18S, 28S, and codon positions of CAD and COI (Song et al. 2018). The ModelFinder Plus algorithm was used to select the best-fit substitution model for each partition. Branch support was evaluated with 1000 ultrafast bootstrap replicates and 1000 SH-aLRT tests. For BI analysis, MrBayes 3.2.7 (Ronquist et al. 2012) was used with a partitioning strategy and substitution models selected by PartitionFinder2 (Lanfear et al. 2017), resulting in seven distinct partitions. Markov chain Monte Carlo (MCMC) analysis was run for 10 million generations across four independent chains, sampling every 1000 generations and discarding the first 25% of samples as burn-in. Convergence was assessed by confirming that the average standard deviation of split frequencies fell below 0.01 and that the effective sample size (ESS) for all parameters exceeded 200. Outgroups were rerooted using species from Protanypodinae (e.g., *Protanypus morio* and *Protanypus caudatus*), Tanypodinae (e.g., *Alotanypus venustus* and *Tanypus* sp.), and Podonominae (e.g., *Podochlus tasmaniensis* and *Podonomopsis evansi*). A full species list is provided in Suppl. material 2.

## Results

### Taxonomy

#### Kaluginia wangi

Taxon classificationAnimaliaDipteraChironomidae

Song & Qi sp. nov.

D7D46BF6-721F-5F55-84B7-D26D0FC655A1

https://zoobank.org/A8BBF8FE-7AD5-405C-86FC-EFB2272143A4

**Figure 1.**
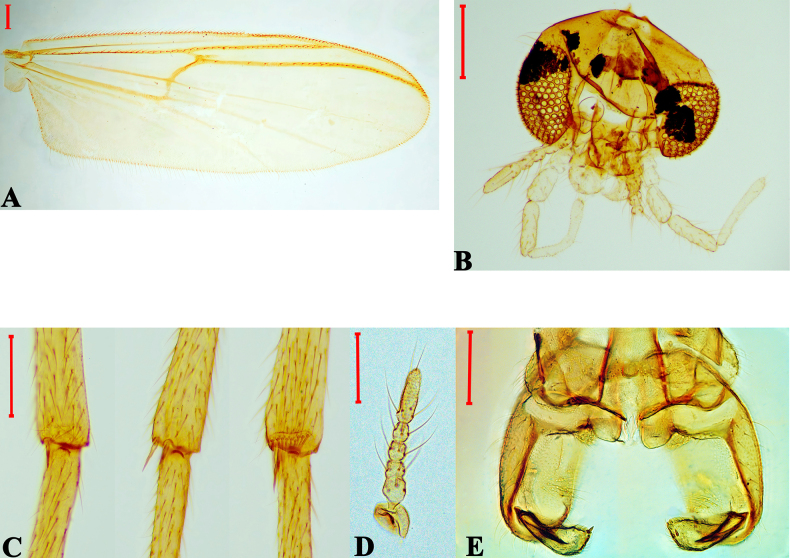
*Kaluginia wangi* sp. nov. adult male. **A**. Wing; **B**. Head; **C**. Tibiae; **D**. Antenna; **E**. Hypopygium. Scale bars: 100 µm.

**Figure 2.**
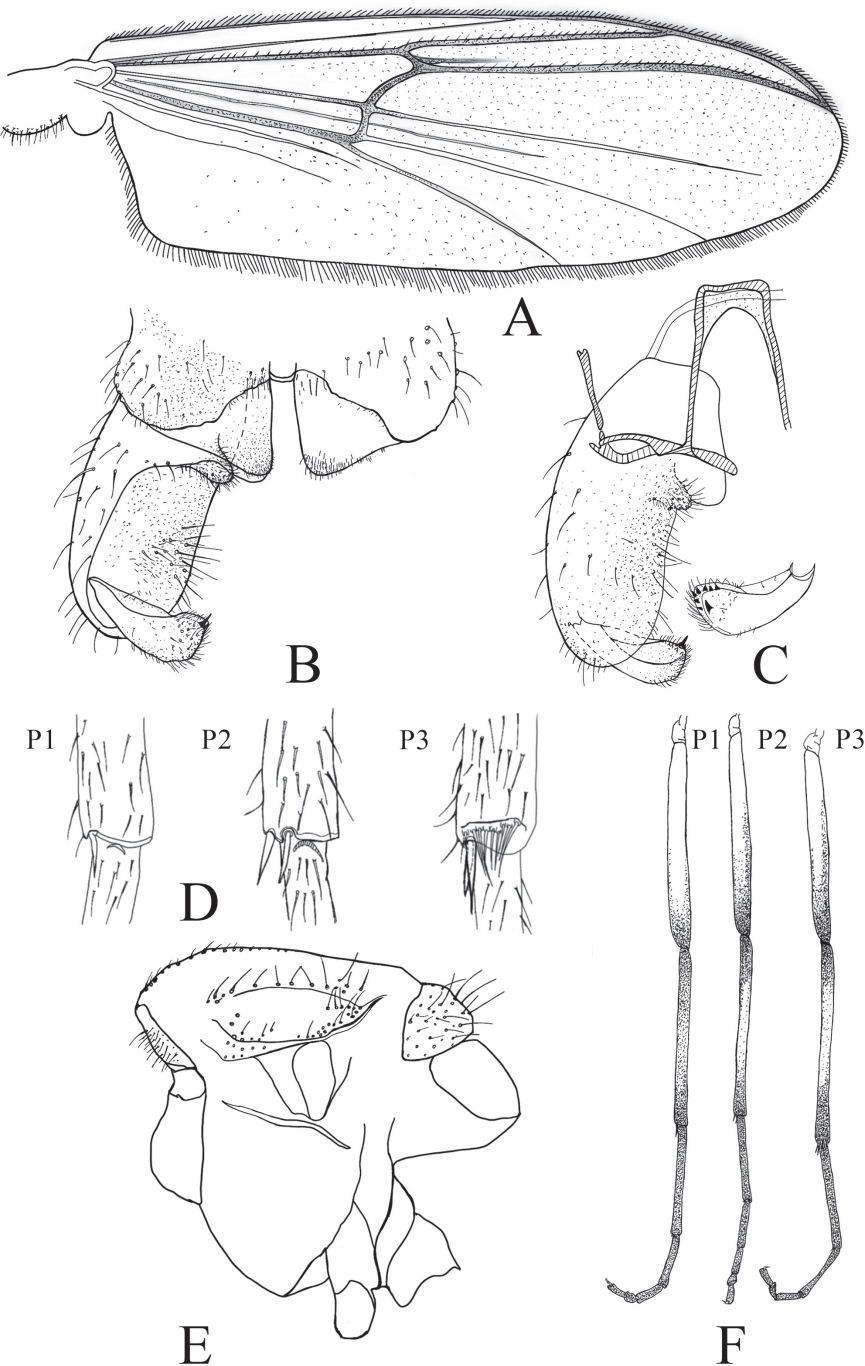
*Kaluginia wangi* sp. nov. adult male. **A**. Wing; **B**. Hypopygium in dorsal view; **C**. Hypopygium in ventral view; **D**. Tibiae; **E**. Thorax; **F**. Legs.

**Figure 3.**
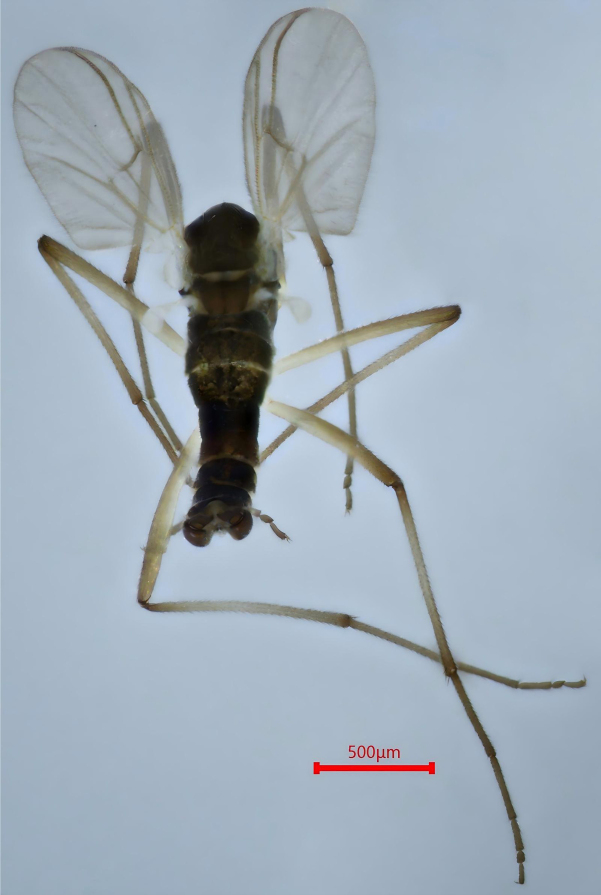
Male adult (holotype, in dorsal view) of *Kaluginia wangi* sp. nov.

**Figure 4.**
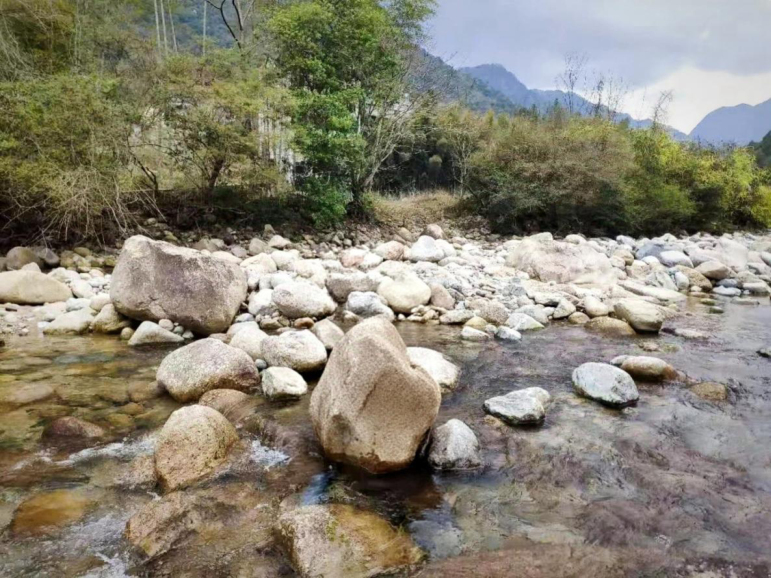
Type locality of *Kaluginia wangi* sp. nov., showing its characteristic montane stream habitat with clear water, a rocky substrate, and densely vegetated banks.

**Figure 5.**
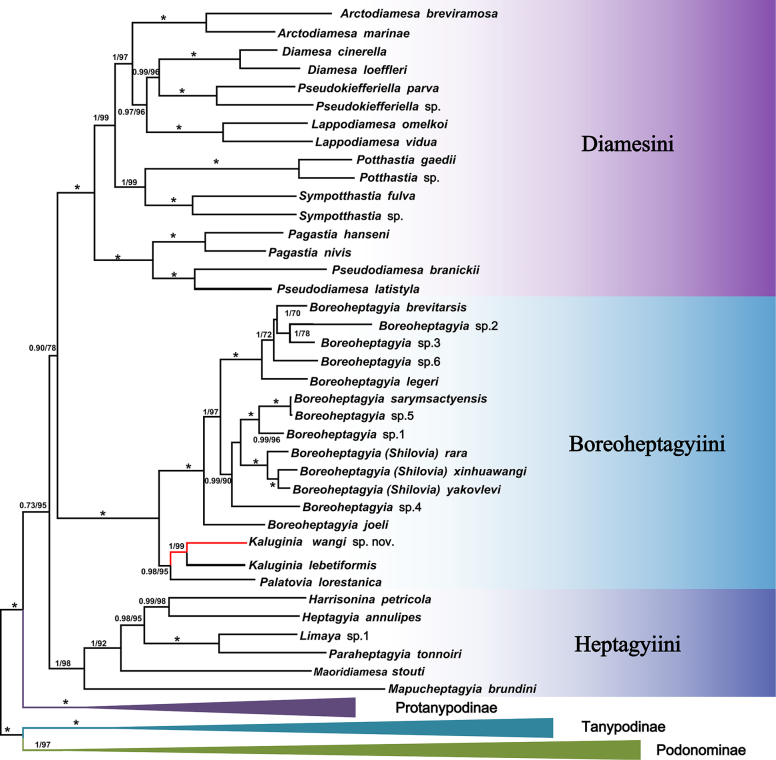
Multilocus phylogenetic tree of Diamesinae. Posterior probability after MrBayes (values > 0.7) and bootstrap support of maximum likelihood (> 70%) are shown, and asterisks (*) indicate full node support.

**Figure 6.**
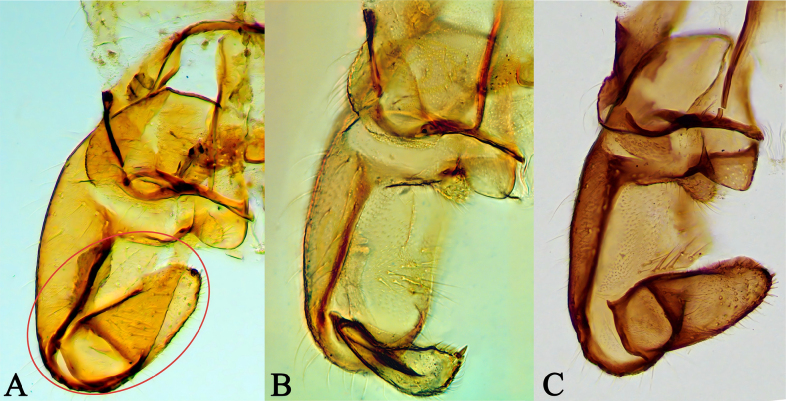
Comparison of male hypopygia. **A**. Holotype, initial preparation (gonostylus folded upwardly with the gonocoxite); **B**. Holotype, after reprocessing; **C**. Paratype.

Figs 1, 2, 3, 4, 5, 6

##### Generic diagnosis.

Genus *Kaluginia* is characterized by the following combination of features: body predominantly dark brown to blackish, antenna with 5 or 7 flagellomeres and reduced plume, eye bare; membrane without setae but with fine punctation, R_4+5_ with setae, R_2+3_ reduced and visible only in basal part, squama fringed, setae in 2–3 irregular rows; antepronotal lobes narrowly joined medially, lateral antepronotals occupy basal 2/3 of lobe, acrostichals short in 1 row beginning near antepronotum, dorsocentrals erect in 1–2 rows, extending from near the humeral area to close to the scutellum, prealars present, scutellum with numerous setae, arranged in 3–4 irregular rows; ta_4_ in all segments are cordiform; tergite IX with a small rounded protuberance as anal point on dorsal surface. Transverse sternapodeme high, almost trapezoidal, with rounded apex. Gonocoxite with a basal lobe and a setose inferior volsella. Gonostylus broad anteriorly, narrowing posteriorly, sometimes twisted or scoop-shaped, apex and inner margin bearing stout megasetae surrounded by microtrichia.

Diagnostic characters. The male imago can be distinguished from known species of the genus by the following combination of characters: antenna with 5 flagellomeres; gonostylus broad anteriorly and narrowing posteriorly, with some twisted angle, and its apex bearing several stout megasetae surrounded by microtrichia; mid and hind ta_1_ each with two pseudospurs.

##### Material examined.

***Holotype***: • adult male, China, Fujian Province, Nanping City, Wuyishan City, near Da’anyuan Drifting (approx. 27.87696°N, 117.86853°E, alt. 490 m), 16 Apr. 2021, ZHONG KH leg., light trap. ***Paratypes***: • 2 males, China, Zhejiang Province, Hangzhou City, Tianmu Mountain, 30.34371°N, 119.45855°E, alt. 459 m, 20 Apr. 2024, ZANG HM leg., light trap.

##### Etymology.

The specific epithet *wangi* is given in honor of Professor Xinhua Wang, in recognition of his fundamental contributions to the systematics of the Chinese Chironomidae.

##### Description.

Adult male (*N* = 3) total length 2.6–2.97, 2.78 mm. Wing length 1.70–2.48, 2.05 mm. Total length/wing length 1.20–1.53, 1.37. Wing length / length of profemur 1.62–2.07, 1.91.

***Colouration*** (Fig. 3). Head dark brown; thorax scutum, scutellum, and postnotum shiny dark brown to black. Abdomen dark brown, legs (Fig. 2F) segments largely brown, but all femora basal 2/3 of femur yellowish, distal 1/3 brown; tibiae are brown at the basal and apical, with a pale-yellow medial portion. Wings greyish-brown.

***Head*** (Fig. 1B, D). The compound eyes are glabrous. Temporal setae include 3–4 inner verticals, 8–9, 8 outer verticals, 3–4 post verticals, postorbitals clearly present, with 4–5 setae. Clypeus with 12–27, 19 setae. Tentorium 124–158, 144 μm long, 19–24, 23 μm wide, Tentorium slightly swollen basally and narrowing evenly from base to apex; palpomere lengths (in μm): 30–43, 35; 32–58, 44; 69–104, 83; 101–148, 123; 146–237, 187. L: 5^th^/3^rd^: 2.11–2.28, 2.22. Head width / palp length 0.98. Antennal pedicel with 2 short setae, antenna with 5 flagellomeres and reduced plume of short setae; number of setae in flagellomeres 1–5, respectively 9; 5; 5; 5; 16. Terminal flagellomere with 2 subapical setae, 50–60 μm long. Flagellomeres 1–5 length (μm): 78–94, 84; 26–30, 28; 27–32, 29; 24–30, 27; 46–58, 53. AR 0.29–0.34, 0.31.

***Wing*** (Figs 1A, 2A). Membrane without setae and with punctation. Anal lobe well developed, fully fringed. VR 0.80–1.23, 0.99. R with 26–42, 32 setae, R_1_ with 24–42, 33 setae, R_4+5_ with 31–42, 35 setae, R_2+3_ clearly visible only in a small portion of the basal part. Wing squama with 15–18 setae, irregularly 2–3 rows. RM/MCu 1.87–2.07, 1.95.

***Thorax*** (Fig. 2E). Antepronotal lobes narrowly joined medially, lateral antepronotals 12–14 setae. Acrostichals 14–16, 15 short, in 1 row, beginning near antepronotum; dorsocentrals 12–15, 13 erect and in 1–2 rows or in 1 group, extending from near the humeral area to close to the scutellum; scutellum with more than 35 scattered setae, arranged in 3–4 irregular rows; prealars 16–26, 19 divided into anterior (6–11, 8 setae), middle (1–4, 2 setae) and posterior sections with 9–11, 10 setae.

***Legs*** (Figs 1C, 2D, 2F). Spur of fore tibia 36–44, 41 μm long; spurs of mid tibia with spurs 42–48, 46 μm and 44–54, 49 μm long; of hind tibia 40–51, 47 μm and 58–71, 63 μm long. Hind tibial fused combs 53–55, 54 μm long, with 9–11, 9 setae. Fore ta_1_ with 1 pseudospur, 21–32, 27 μm long, ta_2_ with 1 pseudospur, 24–31, 27 μm long; mid ta_1_ with 2 pseudospurs, 23–35, 28 μm and 27–36, 31 μm long, ta_2_ with 1–2 pseudospur, 20–30, 26 μm long and 24–34, 29 µm long, ta_3_ with 1 pseudospur, 26 μm long; hind ta_1_ with 2 pseudospurs, 22–34, 29 µm and 29–37, 33 μm long, and ta_2_ with 2 pseudospurs 25–33, 44 μm and 27–35, 31 μm long. Width at apex of fore tibia 65–75, 71 μm, of mid tibia 60–65, 62 μm, of hind tibia 65–73, 69 μm. Lengths (in μm) and proportions for legs in Table 1. ta_4_ in all segments are cordiform, shorter than ta_5_.

**Table 1.** Lengths (in µm) and proportions for legs of *Kaluginia wangi* sp. nov.

	P1	P2	P3
fe	956–1212, 1084	1087–1361, 1254	975–1335, 1143
ti	887–1242, 1066	900–1222, 1061	1004–1353, 1179
ta_1_	546–768, 648	430–605, 519	533–712, 625
ta_2_	261–382, 323	220–305, 263	296–409, 354
ta_3_	140–206, 175	129–183, 156	138–221, 180
ta_4_	65–78, 70	59–72, 67	64–81, 72
ta_5_	107–123, 115	102–125, 113	99–131, 114
LR	0.59–0.62, 0.61	0.49–0.50, 0.49	0.53
BV	3.80–4.40, 4.11	4.42–4.70, 4.57	4.04–4.21, 4.10
SV	3.20–3.61, 3.34	4.12–4.57, 4.30	3.67–3.77, 3.72

Abbreviations used: BV, length of (femur + tibia + ta_1_) / length of (ta_2_ + ta_3_ + ta_4_ + ta_5_); fe, femur; LR, length of ta_1_ / length of tibia; P1, P2, P3, Fore leg, mid leg, hind leg; SV, length of (femur + tibia) / length of ta_1_; ta_1_–ta_5_, tarsomeres 1–5; ti, tibia.

***Hypopygium*** (Figs 1E, 2B, 2C). Tergite IX with 28 setae, bearing a small rounded protuberance on dorsal surface as anal point, laterosternite IX with 11 setae. Transverse sternapodeme is very thick, broadly arched, high, almost trapezoidal, with rounded apex, and 56–62 µm wide. Aedeagal lobe moderately sclerotized, apex attenuated, extending medially over apex of opposite lobe, with apical part smooth. Gonocoxite 290–368, 321 µm long, with well-developed basal plate. inferior volsella covered with setae. Gonostylus 144–174, 161 µm long, broad anteriorly and narrowing posteriorly, with some twisted angle, apex with some stout megasetae, surrounded by microtrichia. HR: 1.85–2.11, 1.98. HV: 2.36.

##### Habitat.

The specimen was collected from a mountain stream in a humid temperate to subtropical montane forest. The streambed consisted mainly of cobbles, with scattered boulders and patches of fine gravel and sand in low-velocity areas. Vegetation along both banks showed distinct vertical stratification: low shrubs near the water, with an overstory of evergreen broad-leaved and some deciduous trees.

##### Distribution.

The new species is currently known from oriental China based on two collection sites: the type locality in the Wuyi Mountain Range (Fig. 4), Fujian Province, southeastern China (one adult male), and an additional site in the Tianmu Mountain, Zhejiang Province, eastern China (two adult males).

##### Molecular phylogeny.

The final concatenated matrix after alignment comprised 42 taxa, with a total length of 3784 bp (18S: 886 bp; 28S: 675 bp; CAD: 744 bp; COI: 1479 bp). The tree topologies inferred from MrBayes and IQ-TREE (Fig. 5) were highly concordant and consistent with the phylogenetic structure reported in Semenchenko et al. (2024). The subfamily Diamesinae comprises three tribes: Heptagyini, Boreoheptagyiini, and Diamesini. Within the tribe Boreoheptagyiini, the newly discovered *Kaluginia wangi* sp. nov. forms a highly supported clade with *Kaluginia lebetiformis* Makarchenko, 1987, indicating a close sister-group relationship. This clade, together with the previously recognized genera *Boreoheptagyia*, *Kaluginia*, and *Palatovia*, constitutes the core evolutionary lineage of Boreoheptagyiini, with strong branch support values across the tribe.

##### Remarks.

The new species and *Kaluginia lebetiformis* share several similarities, including similar leg coloration and texture, as well as similar internal wing structures. However, they can be distinguished by the following characters (Table 2). In the antenna, *K. wangi* has a flagellum composed of five flagellomeres, whereas *K. lebetiformis* has a flagellum with seven flagellomeres. In leg characters, *K. wangi* bears two pseudospurs on each of the mid and hind ta_1_, whereas *K. lebetiformis* has four pseudospurs on the mid ta_1_ and seven on the hind ta_1_. Regarding the hypopygial structure, *K. wangi* has a gonostylus broad anteriorly and narrowing posteriorly, with a twisted angle, and the apex bears several stout megasetae surrounded by microtrichia; in contrast, *K. lebetiformis* is described as having a broad, scoop-shaped gonostylus with 7–8 megasetae along the inner margin.

**Table 2.** Comparison of some relevant morphological characteristics of *Kaluginia lebetiformis* Makarchenko, 1987 and *Kaluginia wangi* sp. nov.

	*K. wangi*	*K. lebetiformis*
Antenna	Flagellum with 5 flagellomeres.	Flagellum with 7 flagellomeres.
Wing	R_2+3_ clearly visible only in a small portion of the basal part.	R_2+3_ reduced, not reaching C.
Thorax	Ac short, in 1 row, beginning near Ap; Dc erect and in 1–2 rows or in 1 group, extending from near the humeral area to close to the scutellum; scutellum arranged in 3–4 irregular rows. Aps with lateral setae present.	Ac present, beginning near Ap; Dc erect and in 1–2 rows; Scutellum with about 50 setae. Aps (ventral and lateral) present; dorsal ones absent.
Legs	Mid and hind ta_1_ each with 2 pseudospurs.	Mid legs with 4 pseudospurs on ta_1_; hind legs with 7 pseudospurs on ta_1_.
Hypopygium	Gc with well-developed basal plate. inferior volsella covered with setae. Gs broad anteriorly and narrowing posteriorly, with some twisted angle, apex with some stout megasetae, surrounded by microtrichia.	Gc with a small, basal protuberance. Gs broad, scoop-shaped with 7–8 megasetae along inner margin.

Abbreviations used: Ac, acrostichals; Ap, antepronotum; Aps, antepronotals; Dc, dorsocentrals; Gc, gonocoxite; Gs, gonostylus; Ps, pseudospurs; Sct, scutellum.

## Discussion

In this study, the identification of the holotype adult specimen collected from Wuyi Mountain, China (Fig. 6A) underwent a process of repeated verification and subsequent revision. During preliminary morphological examination, the initially prepared holotype (Fig. 6A) exhibited the following features: the gonostylus seemingly bifurcates into two unequal branches—a longer one (broad-based, tapering to an apex with a short megaseta) and a shorter one (broad-based, medially constricted, with a rounded apex)—and the flagellum bore five flagellomeres. Based on these characteristics, the specimen was tentatively identified as a potential new genus of the tribe Boreoheptagyiini.

Notably, when the holotype was reprocessed by Dr. Hongqu Tang, a remarkable change in the hypopygial structure was observed (Fig. 6B): the gonostylus appeared broad anteriorly and narrowed posteriorly, with a twisted orientation; its apex bore several stout megasetae surrounded by microtrichia. The bifurcate structure originally seen in Fig. 6A became obscured, while multiple stout megasetae emerged. We presume that the ‘bifurcate’ structure in Fig. 6A may represent hardened internal muscle tissue rather than part of the external morphology (Fig. 6C shows a paratype from this study, which generally resembles Fig. 6A in overall morphology but lacks the bifurcate structure). The appearance of multiple stout megasetae in Fig. 6B is likely attributable to the naturally twisted orientation of the gonostylus, where minor differences in orientation during mounting may result in the masking or exposure of certain structures. By comparing morphological differences in the holotype before and after orientation treatment and contrasting it with other genera within Boreoheptagyiini, we found that the specimen closely resembles the genus *Kaluginia* in morphology. Furthermore, in the phylogenetic tree, it formed a highly supported clade with the known species *K. lebetiformis* (Fig. 5). Consequently, the specimen is identified as a new species of *Kaluginia*, which is described herein as *Kaluginia wangi* sp. nov.

During the mounting process, variations in pressure applied and specimen orientation can lead to different morphological appearances of the same structure, thereby directly affecting the observation and interpretation of diagnostic characters. This verification process demonstrates that accurate species identification and description are highly dependent on well-preserved, properly prepared specimens. This finding highlights the critical importance of meticulous specimen preparation, particularly when dealing with the male hypopygium, which possesses complex three-dimensional structures such as the twisted gonostylus. Subtle procedural differences during mounting—including variations in mounting pressure and minor shifts in orientation—can obscure or distort diagnostic features, subsequently affecting correct taxonomic assignment and even confounding the interpretation of phylogenetic relationships. Therefore, when describing new taxa, multiple meticulous preparations of key specimens, combined with cross-validation using molecular data, are essential to eliminate morphological artifacts and ensure the reliability of taxonomic conclusions.

## Supplementary Material

XML Treatment for Kaluginia wangi

## Additional information

### Artificial Intelligence (AI) use

The authors accept full responsibility for the content of the manuscript, including the disclosure of any use of AI.

No AI tools were used in the preparation of this manuscript.

### Author ORCIDs

Y. Wang https://orcid.org/0009-0002-2541-1327

C. Song https://orcid.org/0000-0002-8981-2296

X. Liu https://orcid.org/0009-0002-3261-1407

X. Qi https://orcid.org/0000-0001-6949-7797

### Data availability

All of the data that support the findings of this study are available in the main text or Supplementary Information.

## Supplementary materials

Supplementary material 1 Primers used for PCR and sequencing in the study Data type docx https://binary.pensoft.net/file/1668194This dataset is made available under the Open Database License (http://opendatacommons.org/licenses/odbl/1.0/). The Open Database License (ODbL) is a license agreement intended to allow users to freely share, modify, and use this Dataset while maintaining this same freedom for others, provided that the original source and author(s) are credited. Yiyi Wang, Chao Song, Xin Liu, Xin Qi

Supplementary material 2 List of species, codes and GenBank accessions used in this study Data type xlsx https://binary.pensoft.net/file/1668195This dataset is made available under the Open Database License (http://opendatacommons.org/licenses/odbl/1.0/). The Open Database License (ODbL) is a license agreement intended to allow users to freely share, modify, and use this Dataset while maintaining this same freedom for others, provided that the original source and author(s) are credited. Yiyi Wang, Chao Song, Xin Liu, Xin Qi
