# Supplementary material for: Lessons from the slide preparation: A new species of Kaluginia (Diptera, Chironomidae) from China
Source: Zookeys. 2026 Jun 4;1281:233–45. doi: 10.3897/zookeys.1281.193732 (PMC13261310; doi:10.3897/zookeys.1281.193732)
Supplement: Supplementary material 1 — Primers used for PCR and sequencing in the study [file zookeys-1281-233_article-193732__-s001.docx]

| **Number** | **Gene** | **Primer** | **Strand** | **Sequence** | **References** |
| --- | --- | --- | --- | --- | --- |
| 1 | 18S rDNA | 18S_ai | Forward | 5’ – CCTGAGAAACGGCTACCACATC – 3’ | Whiting et al. 1997 |
| 2 | 18S rDNA | 18S_bi | Reverse | 5’ – GAGTCTCGTTCGTTATCGGA – 3’ | Whiting et al. 1997 |
| 3 | 28S rDNA | S3660 | Forward | 5’ – GAGAGTTMAASAGTACGTGAAAC – 3’ | Morse and Normark. 2006 |
| 4 | 28S rDNA | A335 | Reverse | 5’ – TCGGAAGGAACCAGCTACTA – 3’ | Whiting et al. 1997 |
| 5 | CAD | 787F | Forward | 5’ – GGDGTNACNACNGCNTGYTTYGARCC – 3’ | Moulton and Wiegmann. 2004 |
| 6 | CAD | 1098R | Reverse | 5’ – TTNGGNAGYTGNCCNCCCAT – 3’ | Moulton and Wiegmann. 2004 |
| 7 | COI–5p | LCO1490 | Forward | 5’ – GGTCAACAAATCATAAAGATATTGG – 3’ | Folmer et al. 1994 |
| 8 | COI–5p | HCO2198 | Reverse | 5’– TAACTTCAGGGTGACCAAAAAATCA – 3’ | Folmer et al. 1994 |
| 9 | COI–3p | s2183 | Forward | 5’ – CAACATTTATTTTGATTTTTTGG – 3’ | Simon et al. 1994 |
| 10 | COI–3p | a3014 | Reverse | 5’ – TCCAATGCACTAATCTGCCATATTA – 3’ | Simon et al. 1994 |

**References:**

Folmer O, Black M, Hoeh W, Lutz R (1994) DNA primers for amplification of mitochondrial cytochrome c oxidase subunit I from diverse metazoan invertebrates. Molecular Marine Biology and Biotechnology 3: 294–299.

Morse GE, Normark BB (2006) A molecular phylogenetic study of armoured scale insects (Hemiptera: Diaspididae). Systematic Entomology 31: 338–349.

Moulton JK, Wiegmann BM (2004) Evolution and phylogenetic utility of CAD (rudimentary) among Mesozoic‐aged Eremoneuran Diptera (Insecta). Molecular Phylogenetics and Evolution 31: 363‐378. <https://doi.org/10.1016/S1055-7903(03)00284-7>

Simon C, Frati F, Beckenbach A, Crespi B, Liu H, Flook P (1994) Evolution, weighting, and phylogenetic utility of mitochondrial gene sequences and a compilation of conserved polymerase chain reaction primers. Annals of the Entomological Society of America 87: 651–701. <https://doi.org/10.1093/aesa/87.6.651>

Whiting MF, Carpenter JC, Wheeler QD, Wheeler WC (1997) The Strepsiptera problem: phylogeny of theholometabolous insect orders inferred from 18S and 28S ribosomal DNA sequences and morphology. Systematic Biology 46: 1–68. <https://doi.org/10.2307/2413635>.
